# *Chlamydia trachomatis ompA* Variants in Trachoma: What Do They Tell Us?

**DOI:** 10.1371/journal.pntd.0000306

**Published:** 2008-09-24

**Authors:** Aura A. Andreasen, Matthew J. Burton, Martin J. Holland, Spencer Polley, Nkoyo Faal, David C.W. Mabey, Robin L. Bailey

**Affiliations:** 1 London School of Hygiene & Tropical Medicine, London, United Kingdom; 2 Medical Research Council Laboratories, Fajara, The Gambia; University of California San Francisco, United States of America

## Abstract

**Background:**

Trachoma, caused by *Chlamydia trachomatis* (*Ct*), is the leading infectious cause of blindness. Sequence-based analysis of the multiple strains typically present in endemic communities may be informative for epidemiology, transmission, response to treatment, and understanding the host response.

**Methods:**

Conjunctival and nasal samples from a Gambian community were evaluated before and 2 months after mass azithromycin treatment. Samples were tested for *Ct* by Amplicor, with infection load determined by quantitative PCR (qPCR). *ompA* sequences were determined and their diversity analysed using frequency-based tests of neutrality.

**Results:**

Ninety-five of 1,319 (7.2%) individuals from 14 villages were infected with *Ct* at baseline. Two genovars (A and B) and 10 distinct *ompA* genotypes were detected. Two genovar A variants (A1 and A2) accounted for most infections. There was an excess of rare *ompA* mutations, not sustained in the population. Post-treatment, 76 (5.7%) individuals had *Ct* infection with only three *ompA* genotypes present. In 12 of 14 villages, infection had cleared, while in two it increased, probably due to mass migration. Infection qPCR loads associated with infection were significantly greater for A1 than for A2. Seven individuals had concurrent ocular and nasal infection, with divergent genotypes in five.

**Conclusions:**

The number of strains was substantially reduced after mass treatment. One common strain was associated with higher infection loads. Discordant genotypes in concurrent infection may indicate distinct infections at ocular and nasal sites. Population genetic analysis suggests the fleeting appearance of rare multiple *ompA* variants represents purifying selection rather than escape variants from immune pressure. Genotyping systems accessing extra-*ompA* variation may be more informative.

## Author Summary

Trachoma is an important cause of blindness resulting from transmission of the bacterium *Chlamydia trachomatis.* One way to understand better how this infection is transmitted and how the human immune system controls it is to study the strains of bacteria associated with infection. Comparing strains before and after treatment might help us learn if someone has a new infection or the same one as before. Identifying differences between disease-causing strains should help us understand how infection leads to disease and how the human host defences work. We chose to study variation in the chlamydial gene *ompA* because it determines the protein MOMP, one of the leading candidates for inclusion in a vaccine to prevent trachoma. If immunity to MOMP is important in natural trachoma infections, we would expect to find evidence of this in the way the strains varied. We did not find this, but instead found that two common strains seemed to cause different types of disease. Although their MOMPs were very slightly different, this did not really explain the differences. We conclude that methods of typing strains going beyond the *ompA* gene will be needed to help us understand the interaction between *Chlamydia* and its human host.

## Introduction

Trachoma is the leading infectious cause of blindness worldwide [1]. Repeated infection by *Chlamydia trachomatis* provokes chronic follicular conjunctivitis (clinically active trachoma), which leads to conjunctival scarring, entropion, trichiasis and ultimately blinding corneal opacification. Trachoma is a major public health problem affecting some of the world's poorest regions. Current estimates indicate 84 million have active trachoma, with 7.6 million visually impaired from trachomatous corneal opacification [2]. The World Health Organization is leading a global effort to control blinding trachoma through the implementation of the SAFE Strategy: Surgery for trichiasis, Antibiotics to reduce the burden of chlamydial infection, and face washing and environmental improvements to limit transmission [3].

Endemic trachoma is caused by 4 of the 19 recognised serovars of *C.trachomatis:* A, B, Ba and C. Serovars are distinguished from each other on the basis of surface variations in the Major Outer Membrane Protein (MOMP). As the main antigenic target for strain specific humoral immunity to *C.trachomatis,* MOMP has been considered a vaccine candidate [4]. MOMP is encoded by the *ompA* gene, which contains four variable segments (VS) interspersed between five conserved segments (CS). Comparative genome sequence analysis has indicated considerable variation in *ompA*, possibly driven by host immune pressure, and the study of *ompA* variants may therefore be informative in disease settings [5],[6] Originally serovars were distinguished according to their recognition by panels of patient sera, however the *ompA* sequence motifs for each serovar have now been well characterised. Organisms assigned to a serovar group on the basis of their *ompA* sequence are referred to here as genovars.

*OmpA* genotyping has been used previously to investigate *C.trachomatis* infections in trachoma endemic populations [7]–[14], usually with the goal of better understanding *C.trachomatis* transmission. However the analysis of o*mpA* sequence variation is also relevant to the utility of MOMP as a target for chlamydial vaccine development. In genital infections caused by *C.trachomatis* D-K genovars, evidence that genovar and strain variants associate with clinically important differences in the biology of infection is marginal [15], and has not been described in human ocular infection. Here we analyse *ompA* genotypic diversity before and two months after mass antibiotic treatment of trachoma in Gambian villages [16],[17].

## Methods

### Ethical Permission

The Gambian Government/Medical Research Council Joint Ethics Committee (SCC 856) and the London School of Hygiene and Tropical Medicine Ethics Committee approved the study. All subjects, or their guardians, gave written informed consent, or witnessed consent by thumbprint where appropriate.

### Clinical Assessment

This study was conducted in 14 trachoma endemic Gambian villages, located within a defined geographical area [16],[17],[18]. The villages were surveyed and a population census was conducted. Individuals normally resident in the study area for at least 6 months of the year were enrolled. At baseline the entire available population was examined for signs of trachoma and classified using the WHO Trachoma Grading System [19]. A swab sample was collected from the upper tarsal conjunctiva of each subject for DNA isolation and kept cool until frozen at −20°C later the same day. Swabs of fresh nasal discharge were collected.

### Antibiotic Treatment

Following baseline clinical assessment, all participants were offered antibiotic treatment. Adults and children over 6 months old were given a single oral dose of azithromycin (20mg/kg up to a maximum of 1g). Infants under 6 months were given tetracycline eye ointment (twice daily, 6 weeks). All villages were examined and treated within a 9 day period [17].

### Follow-up

Two months after baseline assessment and antibiotic treatment, participants were re-examined, and conjunctival and nasal discharge samples again collected. Between these two time points, the census was updated weekly, together with records of destination and duration of travel and of the presence of any external visitors.

### *Chlamydia trachomatis* detection

DNA was extracted from the swabs and tested using the Amplicor CT/NG kit (Roche) [16]. Amplicor extracts from specimens with detectable *C.trachomatis* DNA were further purified and concentrated using the QIAamp DNA Mini Kit (Qiagen) [16]. Infection load was estimated by quantitative real-time PCR for the chlamydial *ompA* gene using a previously described method [20].

### *ompA* Sequencing

Sequencing of *ompA* used primers spanning VS1-4 and sequences were comfirmed by a second sequencing pass. A 1076bp fragment was amplified using primers 87: 5′ - TGA ACC AAG CCT TAT GAT CGA CGG - 3′ and 1163: 5′ - CGG AAT TGT GCA TTT ACG TGA G - 3′. If no amplified product was visible on an agarose gel, nested PCR was performed, with primers 87 (above) and 1059: 5′ - GCA AGA TTT TCT AGA TTT CAT C - 3′ used to amplify a 972bp target sequence. PCR products were purified using the QIAquick PCR purification kit (Qiagen) and sequenced using BigDye Terminator Cycle Sequencing Ready Reaction kit V3.1(Applied Biosystems) with outer primers 97: 5′ - CTT ATG ATC GAC GGA ATT TTC TAT GGG - 3′ and 1047: 5′ - GAT TTT CAT GAT TTC ATC TTG TTC AAC TG - 3′. Sequencing with inner primers 608: 5′ - CTC TCT GGG AAT GTG GGT GT - 3′ and 627: 5′ - ACA CCC ACA TTC CCA GAG AG - 3′ was performed to close sequencing gaps. Sequences were edited and aligned using DNA*DNASTAR 5.07 (DNASTAR), with HAR 13 (NC_007429) as genovar A reference and M33636 for genovar B. Here, a genotype denotes an *ompA* sequence variant differing from the *ompA* reference sequence or from another variant by one or more single nucleotide substitutions, and is identified using the letter of its genovar and an arbitrary number.

### Analysis

Data were analysed in Stata 9.0, with differences in loads per genotype examined using a two tailed t-test on logtransformed loads. Sequence alignments were imported into DNAsp4.00 and Tajima's D value calculated [21],[22]. P-values for each D test were calculated using 10,000 coalescent simulations without the presence of recombination to calculate the proportion of D values generated which were greater than the observed D value. D* and F* indices were calculated as further tests of the neutrality of mutations [23].

## Results

### Study population

1319 (83%) of 1595 people enumerated at baseline were examined, sampled and treated. At two-months 1344 (85%) were examined and sampled. The overall prevalence of active trachoma in children <10 years was 16% before and 12% two months after treatment, with marked variations in prevalence between villages [16].

### *C. trachomatis* Infection

The prevalence of *C.trachomatis* infection was 7.2% (95/1319) before treatment and 5.7% (76/1344) two months after treatment. Of individuals infected at baseline, 30% were still infected two months after treatment and of those infections detected at two months 36/66 (59%) occurred in subjects uninfected at baseline (Table 1). Most infections (74/76; 97%) detected two months after treatment were in two villages. Almost all residents of these two villages travelled *en masse* to a religious festival one month after the treatment. This travelling event was very strongly associated with infection at two months [16]. In contrast, in the other 12 study villages all cases of *C.trachomatis* infection found at baseline had resolved by two months and there were only 2 new cases of infection in previously uninfected individuals.

**Table 1:** Comparative ocular *C. trachomatis* infection status (by Amplicor) before and two months after antibiotic treatment amongst all those tested at both time points.

	2 months		Total
	+	−	
**Baseline**			
**+**	27	60	87
**−**	39	1030	1069
**Total**	66	1090	1156

(+ infected, − not infected).

### *ompA* genotypes

77/95 (81%) baseline and 64/76 (84%) two-month ocular *C.trachomatis* samples yielded sequence data. On both occasions sequence data were obtained from all 5 Amplicor-positive nasal specimens. 73 (95%) of the baseline ocular sequences were genovar A and 4 (5%) were genovar B. Overall, ten separate genotypes were identified; 8 genovar A and 2 genovar B. Sequence variation compared to reference strains is shown in Table 2. For most genotypes single nucleotide polymorphisms (SNPs) resulted in amino acid changes in the variable sequence domains of MOMP. Within genovar A baseline sequences, there were eight polymorphic sites, of which five contained singletons (SNPs found only in a single isolate). Tajima's D value for baseline genovar A sequences was −1.06, revealing trend towards an excess of rare mutations, (p = 0.16). This was supported by significantly negative D* and F* indices, indicating an excess of singleton mutations amongst genovar A sequences (−2.59; P = 0.02 and − 2.45; P = 0.02 respectively). Only four genovar B sequences were found, therefore frequency based analyses could not be performed. However, addition of these four sequences to the genovar A sequences for calculation of an overall Tajima's D value revealed a significant excess of rare mutations within the baseline dataset as a whole (D = −1.76 ; p = 0.018).

**Table 2:** *Chlamydia trachomatis* strains identified in this study.

Genovar A strain	VS1	VS1	CS2	CS2	VS2	VS2	CS4	VS4	VS4	Genovar B strain	VS2
Nucleotide	278	289	304	353	505	523	790	991	1020		511
Ref A (HAR13)	T (V)	G (E)	G (A)	C (A)	G (G)	A (I)	A	A (T)	G	Ref B (M33636)	G(G)
**A 1**	.	.	.	.	.	.	G	.	.	B1	A (G→S)
**A 2**	.	.	A (A→T)	.	.	C (I→L)	G	.	A	B2	
**A 3**	.	.	A (A→T)	.	.	C (I→L)	G	.	.		
**A 4**	.	.	.	.	.	.	G	G (T→A)	.		
**A 5**	A (V→E)	C (E→Q)	.	.	A (G→S)	.	G	.	.		
**A 6**	.	.	A (A→T)	T (A→V)	.	C (I→L)	G	.	A		
**A 7**	.	.	A (A→T)	.	.	.	G	.	A		
**A 8**	.	.	.	.	.	.	G	.	A		

Only single nucleotide polymorphisms and their locations are shown. Reference strains are *C. trachomatis* HAR 13 (genovar A) (NC_007429) and *C. trachomatis* M33636 (genovar B). Letters in parenthesis represent the amino acid and any resulting change. Genotype B2 is identical to the reference strain. (VS, variable sequence. CS, conserved sequence.)

### Genotype distribution

Genotype frequencies are presented in Table 3. The dominant strain, A2, accounted for 74% of baseline ocular isolates. All other strains, except A1, were detected in only a few individuals. The 14 villages contained 79 family compounds (fenced areas inhabited usually by the members of one extended family). 16 (20%) contained subjects infected at baseline. Seven compounds contained multiple strains; three of which had 3 strains and one 5 different strains. Obvious environmental risk factors which might explain this concentration of diversity were not identified: however the latter compound had an unusually high proportion of its children attending the local primary school (7/25; 28%) compared to (30/773: 4%) in the study area generally.

**Table 3:** Frequency of *C. trachomatis* strains present at baseline and 2 months, subdivided by site of collection. Numbers in parenthesis are %.

	Ocular		Nasal	
Genotype	Baseline (n = 77)	2 Months (n = 64)	Baseline (n = 5)	2 Months (n = 5)
**A1**	10 (13)	5 (8)	-	1 (20)
**A2**	57 (74)	58 (90.5)	4 (80)	3 (60)
**A3**	2 (2.5)	-	1 (20)	-
**A4**	1 (1.3)	-	-	-
**A5**	1 (1.3)	1 (1.5)	-	-
**A6**	1 (1.3)	-	-	-
**A7**	-	-	-	1 (20)
**A8**	1 (1.3)	-	-	-
**B1**	1 (1.3)	-	-	-
**B2**	3 (4)	-	-	-

### Genotypes following treatment

At two months post-treatment only three strains A1, A2 and A5 were found. The A2 proportion increased to 90%. Rare strains had mostly disappeared. In 23 individuals ocular samples yielded sequence data at both time points. 18 (78%) of these had the same strain at both timepoints: 3 A1, 14 A2 and 1 A5 (the only example of A5 at either timepoint). 5 (22%) showed a change in genotype: from A1 to A2 in three cases, from A3/A4 to A2 in one case each. 34/35 (97%) newly infected individuals at two-months had the A2 genotype.

### Genotypes and infection load

Infection load data from this population has been previously described [16],[17]. Geometric mean infection loads for strains A1 and A2 were compared by unpaired, two-sided t-tests on logarithmically transformed data. Chlamydial load was significantly higher in A1 infections before mass treatment: geometric mean for A1 5809 copies (95% CI 374–90189) (n = 6) and for A2 92 copies (95% CI 59–144) (n = 14) (p<0.0001). Similarly, after mass treatment geometric mean for A1 was 343 copies (95% CI 42–277663) (n = 3) compared to 115 copies (95% CI 66–202) (n = 19) (p = 0.0021). At both baseline and two-months, subjects infected with A1 were more likely than those infected with A2 to have clinically active disease: baseline: 7/10 vs 6/57 (RR = 6.65, χ ^2^ = 15.63, p<0.0001); two-months 3/5 vs 7/58 (RR = 4.97 p = 0.025 2-tailed Fisher's Exact Test). We have previously found that infected individuals with clinical signs of trachoma have higher chlamydial loads than those without signs [16],[17]. These analyses are not adjusted for potential clustering by village: however A1 only occurred in one village (village 3).

### Nasal genotypes

*C trachomatis* was detected in nasal samples from 5/58 subjects at baseline, and from 5/54 at two months. In seven subjects *ompA* sequence was determined in both ocular and nasal samples at the same time point: 5/7 (71%) had different genotypes at the two sites: A1(ocular)/A2 (nasal) in three cases, with A1(ocular)/A3(nasal) and A2 (ocular)/A7(nasal) in one each. Differing genotypes were found in all four individuals in whom baseline ocular and two-month nasal *ompA* sequence were both determined, and in the two individuals in whom baseline nasal and two-month ocular *ompA* sequences were both determined.

## Discussion

In this study, 972 bp sequences comprising almost the entire *C.trachomatis ompA* gene were determined in samples from infected individuals in a trachoma endemic area. Previous trachoma studies have sequenced primarily VS regions: variation in the interspersing ‘conserved’ segments is recognised but not usually examined at the pathogen population level. All variants were confirmed with double pass sequencing methods: dubious calls on the chromatogram were all clarified by resequencing. We discuss the utility of o*mpA* genotyping for determining the existence and nature of selection pressure on the locus, for examining whether variants affect the features of infection or disease, and for distinguishing causes of reemergent infection after treatment.

Ten *C.trachomatis* genotypes were identified at baseline. Excepting B2, these differed from strains previously sequenced from The Gambia and elsewhere [7]–[11],[13]. Before treatment most (87%) infections were one of two strains (A1 and A2). Six of the minority genovar A strains had SNPs resulting in amino acid changes within variable segment domains. A similar pattern of a few dominant strains with several other strains present at low frequency has been described previously [7],[10],[14]. The variety of strains in this limited geographical area might suggest that new strains are regularly introduced through mixing with other populations or alternatively that the emergence of new variants is promoted by pressure from the human immune response. To test this frequency based analyses of polymorphism were carried out.

Population genetic analysis of baseline genovar A *ompA* sequences showed negative Tajima's D, Fu and Li's D* and F* statistics, suggesting that in this environment novel genovar A *ompA* mutations are being eliminated from the population. Despite this, the location of some of the polymorphic sites is intriguing. In genotype A5 the neutralizing antibody epitope which defines serovar A (^70^DVAGLEK^76^) is significantly altered (^70^D**E**AGL**Q**K^76^): previously we noted significant alteration in close proximity to this epitope (^69^(S→**R**)DVAGLEK^76^) in strains which subsequently failed to establish themselves in the community [10]. One would expect that novel mutations which allow immune evasion offer the pathogen a selective advantage (at least while these strains remain uncommon), and ought to spread through the pathogen population until they reach intermediary frequencies. The excess of rare mutations observed at baseline therefore does not support the hypothesis that *ompA* polymorphisms are maintained within this population by immune selection pressure. Instead it implicates either ongoing negative selection (where most mutations are deleterious and removed from the population by purifying selection) or a recent selective sweep (whereby a single haplotype has reached fixation within the population, driving out diversity at the locus). Few studies have applied population genetic methods to analyse selection of *C.trachomatis* genes, but they have similarly generated little evidence that *ompA* is under immune selection pressure: both cross sectional studies of genovar A *ompA* sequences from Tanzania and sequence analysis of genital *Ct* genovars have found similar evidence of purifying selection in *ompA* 11,24 These data and the existence of individuals within trachoma endemic communities who are often or repetitively infected with the same *ompA* genovar lead us to question whether the *ompA* locus is a target of selective pressure in trachoma populations, and consequently whether targeting MOMP will lead to an effective vaccine.

Strain-specific differences affecting infection or disease manifestations are described in genital chlamydial infection, but not previously in trachoma. On both occasions strain A1 was associated with clinical signs of active trachoma and with higher mean infection loads to a greater extent than A2, but it was less common in the community and so not necessarily a more successful pathogen. The sampling method used here has been shown elsewhere to give adequate yields of host RNA [18], but the infection loads were not standardised, for example against host DNA yield in the sample. In natural infections the number of cells sampled, the proportion of host cells which are infected and the state of the chlamydial developmental cycle within them will all affect the measured load, and the best way to standardise the measurements is not clear. A1 and A2 might amplify differently by PCR, although there was no support for this suggestion in the amplification of standards, and no variation affecting primer binding sites. Differences in sampling, in PCR amplification or in the infection/disease course within the sampled individuals might explain this observation, or alternatively it could result directly or indirectly from variation in *ompA* .

Three differences exist in the *ompA* sequence of A1 and A2, of which two cause non-synonymous amino acid substitutions. These might alter the conformation of MOMP or have direct effects on ‘fitness’, transmission or the host response. The G→A mutation at position 304 introduces a cleavage motif for cathepsin-L, which generates of peptide fragments for antigen presentation [25],[26]. Whether peptide fragments of A1 and A2 MOMP are therefore presented differently during the generation of adaptive cellular immunity is unknown. Alternatively, strain differences might be unrelated to *ompA* itself but reflect linkage between *ompA* genotype and polymorphism(s) elsewhere on the chlamydial chromosome leading to differences in fitness or metabolic advantage. Trachoma strains may differ in their laboratory properties, and a recent study found differences in *in vitro* growth rate, interferon-γ sensitivity and virulence in non-human primates [27], attributable to variation affecting 22 open reading frames(ORFs) in addition to *ompA*. Both clinical differences between strains, and the purifying selection at the *ompA* locus could result from variation or selection pressure at linked chlamydial ORFs.

Following mass antibiotic treatment there was a modest reduction in the prevalence of infection [17]. Only 3 of the original 10 genotypes were still present. Most (90.5%) of these infections were with A2, and almost all in two villages (1 and 3 in Table 4), in which the prevalence of infection actually increased [17], with strains A1 and A2 continuing to dominate. New infections, 97% with strain A2, were strongly associated with travel to a festival in Senegal, at which over a million people from the region congregated in basic conditions, where the opportunity to acquire ocular *C.trachomatis* infection was probably considerable. These data suggest that a remarkable re-infecting exposure to strain A2 occurred in the treated subjects during this event. The persistence of the common A1 or A2 strains in 17 individuals in these villages could be due to treatment failure or to reinfection facilitated by the same unusually effective environment for *C.trachomatis* transmission. Genotyping provides some evidence that antibiotic treatment was not 100% effective, as strain A5 was found twice, but in the same individual both before and after treatment, strongly suggesting primary treatment failure. Nevertheless antibiotic treatment cleared all baseline infections in the other 12 villages [17].

**Table 4:** Clinical activity, infection (Amplicor) and genotypes by village and timepoint.

	BASELINE				2 MONTHS			
Village	Examined	TF/TI	CT+	Genotypes	Examined	TF/TI	CT+	Genotypes
**1**	142	25	20	A2(17) A5(1)	145	21	19	A2(11) A5(1)
**2**	97	2	0	-	97	2	2	A2(2)
**3**	115	18	41	A1(10) A2(19) A3(2) A4(1)	105	16	55	A1(5) A2 (45)
**4**	255	14	8	A2(5)	277	9	0	None
**5**	107	6	0	-	126	10	0	None
**6**	39	6	2	A2(1) B2(1)	37	1	0	None
**7**	86	0	1	-	83	0	0	None
**8**	83	3	1	-	77	6	0	None
**9**	23	0	0	-	21	1	0	None
**10**	60	4	0	-	57	2	0	None
**11**	97	5	0	-	93	1	0	None
**12**	103	14	0	-	98	5	0	None
**13**	53	0	0	-	64	1	0	None
**14**	58	6	22	A2(14) A6(1) A8(1) B1(1) B2(2)	64	4	0	None

For each village, ‘Examined’ is the number of people examined ‘TF/TI’ is the number of individuals (all ages) with active trachoma, and ‘CT+’ the number of those whose ocular swabs tested positive by Amplicor. The numbers bracketed after the genotype indicate the number of times it appeared: A2 (14) denotes 14 samples contained genotype A2.

The surprising demonstration of discordant genotypes in concurrent ocular and nasal samples may imply that these two mucosal surfaces function as distinct sites of infection, despite direct communication via the nasolacrimal duct. This could result from differences in the time course of infection or in the route of inoculation. Autoreinfection of the conjunctiva from extraocular sites such as the nasal mucosa has been suggested, however, a study from Tanzania did not support this hypothesis [28]. Here, the limited nasal genotyping data does not provide support significant transmission between eye and nose.

Our study illustrates the use and limitations of *ompA* sequence data in the molecular epidemiology of *C.trachomatis* infection. The pattern of ompA sequence diversity remains intriguing and inconsistent with immune selection pressure. Typing systems including other polymorphic loci may lead to better elucidation of key events in ocular *C*.*trachomatis* infection. An ongoing extended longitudinal study of *C.trachomatis* genotypes might better define the population dynamics, and determine implications for the long-term success of mass treatment [14].
